# Practical contribution of women development army on growth monitoring and promotion service at Dembya and Gondar Zuria districts, Central Gondar Zone, North West Ethiopia: a community based mixed study

**DOI:** 10.1186/s12887-023-04124-9

**Published:** 2023-06-23

**Authors:** Esmael Ali Muhammad, Melkamu Tamir Hunegnaw, Kedir Abdela Gonete, Netsanet Worku, Kasahun Alemu, Zegeye Abebe, Tigist Astale, Getnet Mitike, Aysheshim Kassahun Belew

**Affiliations:** 1 Department of Human Nutrition, Institute of Public Health, University of Gondar, Gondar, Ethiopia; 2 Department of epidemiology and biostatistics, Institute of Public Health, University of Gondar, Gondar, Ethiopia; 3 International Institute for Primary Health Care -Ethiopia, Addis Ababa, Ethiopia; 4 Flinders Health and Medical Research Institute, College of Medicine and Public health, Flinders University, Adelaide, Australia

**Keywords:** Practical contribution, Women development army, Growth monitoring, Child health, North West Ethiopia

## Abstract

**Background:**

The United Nations’ Sustainable Development Goal (SDG)-2 aims to eliminate child hunger or end all forms of child malnutrition by 2030. To achieve this goal the cost-effective method is the implementation of growth monitoring and promotion service with the contribution of Women Development Army (WDA) as community volunteers. However, According to the data, the program’s implementation varies throughout the country and lack of evidence on the practical contribution of the WDA to enhancing child nutritional care outcomes. Therefore this study aimed to determine practical contribution of WDA and associated factors on growth monitoring and promotion service in two rural districts of central Gondar zone, Northwest Ethiopia.

**Methods:**

A community based mixed study was conducted from March 6 to April 7, 2022 among 615 Women Development Army. Multistage sampling technique was used to select study participants. A structured questionnaire was used to collect quantitative data and in-depth interview were used to generate qualitative information. Qualitative data were coded and grouped and discussed using identified themes. Binary logistic regression was fitted, odds ratio with 95% confidence interval was estimated to identify factors of practical contribution of WDA and qualitative data was analyzed using thematic analysis.

**Results:**

In this study practical contribution of WDA on growth monitoring was 31.4% (95% CI: 28.0-35.3%). Having GMP training (AOR = 4.2, 95%CI: 1.63, 10.58), regular community conversation (AOR = 6.0, 95%CI: 3.12, 11.54), good knowledge about GMP (AOR = 2.1, 95%CI: 1.17, 3.83) and not having regular schedule of GMP service in the area (AOR = 0.04, 95%CI: 0.02, 0.09), were statistically significantly associated with practical contribution of growth monitoring. During in-depth interview, lack of training, low motivation or commitment among WDA and low communication between WDA and health extension workers were mentioned among the problems faced during growth monitoring service.

**Conclusion:**

In this study, practical contribution of growth monitoring among WDA was low. GMP training regular community conversation, knowledge about GMP and regular schedule of GMP service in the local area were significantly associated for practical contribution of growth monitoring service. Lack of training, low motivation or commitment among WDA and low communication between WDA and health extension workers were reasons for did not contribute effectively for GMP service. Therefore, giving training for WDA and improving community conversation at kebeles level are important to improve GM service. .

## Background

The United Nations’ Sustainable Development Goal (SDG)-2 aims to eliminate child hunger or end all forms of child malnutrition by 2030 [1]. Globally, the 2020 report shows that there are 144 million and 47 million under-five children are stunted and wasted, respectively, of these more than half lived in Asia, and two out of five lived in Africa [2]. In addition, globally, about 45% of deaths among under five children are related to malnutrition, with low- and middle-income countries being the most affected regions [3], and malnutrition is the most serious health issue in developing countries [4, 5].

These evidences suggested the serious public health importance of poor child growth and development in low- and middle-income countries [6, 7]. Malnourished children have impaired cognitive capacity, which thereby negatively affects their school performance and future productivity [8–10]. Poor social development capacity is commonly reported in undernourished children [11–13]. The burden of malnutrition costs the global economy up to US$3.5 trillion per year [14].

Despite a significant reduction over the years, malnutrition in Ethiopia is still a major public health problem, with devastating consequences for short-term survival, long-term well-being, and socioeconomic inequality [15, 16]. According to a 2019 Ethiopian Mini Demographic and Health Survey report, 37%, 21%, and 7% of children under the age of five are stunted, underweight, or wasted, respectively [17]. Moreover, the impact of malnutrition has a serious and lasting on the development, economy of the nation, and medical care of individuals and their families [18–20].

To avert this problem, Growth monitoring and promotion (GMP) service is implemented to improve and maintain nutritional and health status of children [21, 22]. It is organized in a community as an outreach service or in a health facility using Women Development Army (WDA) as community volunteers [23]. WDA is a structural arrangement that have a sort of structure and approach. Ethiopia established this approach in 2010 by formulating a one-to-five and one-to-thirty network of woman development teams (WDTs) within the same neighborhood. woman are selected to lead the families because she is knowledgeable about 16 different health extension initiatives and actively practicing those packages [24]. A family is recognized as a model family if they successfully implemented all 16 Health Extension packages (HEP). The implementation of health extension package components like growth monitoring and promotion (GMP) in every family who had young children of less than 2 years [25].

In the last two to three decades, GMP has grown in popularity and is now used in more than 80 nations. Currently, 154 nations, including Ethiopia, use GMP as a crucial component of primary healthcare to improve children’s nutritional status [26]. A research done in Ethiopia and South Africa revealed that health professionals did not implement GMP as suggested, for instance in Ethiopia only 58.4% of the health facilities implemented GMP, despite the International Children’s Emergency Fund of the United Nations’ recommendation for 100% GMP coverage [27, 28].

In order to improve the nutritional status of under five children, the Ethiopian government adopted a strategy involving the Women Development Army (WDA) in Community Based Nutrition (CNB) in 2010 to improve GMP service utilization [24]. Despite this, growth monitoring and promotion services had no significant improvement compared with maternal services after the introduction of WDA in the country. For instance, antenatal care (ANC) increased from 28% to 2005 to 62% in 2016, institutional delivery increased from 5% to 2005 to 26% in 2016, and postnatal care also increased from 5% to 2005 to 17% in 2016 [29, 30].

These figure showed that once the WDA structure was implemented in the nation, there was no sufficient evidence on the practical contribution of WDA to enhancing child nutritional care outcomes. Furthermore, the program’s implementation varies across the region of the country. For example, according to EDHS, 2016 reports Amhara region had the greatest levels of stunting compared with other regions of the country. Therefore this study aimed to determine practical contribution of WDA on growth monitoring and promotion service in two rural districts of central Gondar zone, Northwest Ethiopia.

## Methods

### Study design and setting

Community based mixed study was conducted from March 6 to April 7, 2022 in central Gondar zone, Gondar Zuria and east Dembya districts Northwest Ethiopia. It is located 732 km from Addis Ababa (capital of Ethiopia) in the North West direction. Both districts have one primary hospital, 9 public health centers and 42 health posts. There are 96 health extension workers and 1,260 Women Development Army.

### Study population

The source populations were all Women Development Army who assists health extension workers on GMP and community based nutrition at kebeles level of each destircts with health extension workers, whereas, the study populations were all Women Development Army who doing GM in selected kebeles. For qualitative component of the study 7 participants (3 WDA, 2 Health extension workers and 2 supervisors were enrolled.)

### Inclusion and exclusion criteria

The inclusion criterion of this study was all Women Development Army working GM and community based nutrition at kebeles level with health extension workers at the selected kebeles. The exclusion of this study was Women Development Army working less than 6 months.

### Sample size determination

A single population proportion formula was used to determine the sample size based on the following assumptions. Since there is no study done in the country previously, we considered the proportion of GMP practice was 50%, with 95% CI, 5% margin of error and 10% non-response. Finally 634 sample sizes were obtained by considering 1.5 design effects. For qualitative part of the study, seven participants were selected purposively for in depth interview.

### Sampling procedure

There are 42 kebeles (small administrative units) in the two districts. By using simple random sampling, 21 kebeles, 15 kebeles from Gondar zuria district (Bahariginb, Jayra, Tsion, Chinchaye, Degola, Chihra, Mantero, Dinzaz, Laye, Zengaj, Lamba, Gomengie, Dasmariam, Minziro, and Tachtseda), and 7 kebeles from Dembya district (Robit, Girargie, Aymba,Chilo, Gurambabata, Salgi Jankura) were selected from the two districts, and then all Women Development Army who were directly involved in GMP and community-based nutrition were included in the study.

For qualitative one, a well-structured question was used as a guideline to conduct in-depth interview. The purpose of the in-depth interview was to obtain general qualitative information on the importance and their practical contribution of GMP, and problems encountered during the process and ways of improving GMP services.

#### Data collection

Quantitative data were collected using an interview administered questionnaire, which contains socioeconomic, knowledge, and practise-related characteristics. Four BSc nurses participated during the data collection period. First, 21 kebeles were selected by using a simple random sampling technique from the two districts, and then all the Women Development Army in the selected kebeles were included in the study.

For qualitative data, an in-depth interview was used to obtain the views of the Women Development Army, health extension workers, and supervisors about GMP and the contribution of the Women Development Army in the local area.

#### Operational definitions

The dependent variable for this study was the practical contribution of WDA to growth monitoring and promotion services, defined as the practise and activities of WDA on growth monitoring and community-based nutrition programmes by assisting the health extension workers during the programme. To declare the good practical contribution of WDA, they answered above the median value of the nine practical-related questions.Good knowledge about growth monitoring; if Women Development army correctly answered and scored above the median value of the seven knowledge assessment questions.

#### Data quality control

A two-day training was given for data collectors and supervisors to maintain the quality of the data and how to approach participants. The completeness, accuracy, and consistency of the collected data were checked every day. The pretest was done on 65 WDAs from non-selected districts.

### Data analysis

For the analysis, the data were cleaned and entered into EPI-Info version 7, then exported to SPSS version 25. The results of descriptive statistics and cross tabulation were presented using text, tables, and graphs. In order to select the factors associated with the dependent variable, logistic regression was fitted. In the multivariate analysis, associated variables with a p-value of less than 0.2 were included. Finally, variables that were statistically and significantly associated with the outcome variable were declared using a 95% CI and a p-value of 0.05.

In-depth interviews were used to collect qualitative data, which was then thoroughly transcribed and translated into English, and thematic analysis was used. Following a series of linked procedures, such as reading, coding, presenting, reducing, and interpreting, the data-analysis process was carried out. The data was initially coded after carefully reading the transcripts. Once all the data had been gathered, it was displayed and reduced at a desk.

## Results

### Socio-demographic characteristics of the health development army

More than two-thirds 418 (68.0%) of the health development army were in the age groups of 31–45 years old. The 531(86.3%) of the respondents were married. About two-thirds 391(63.6%) of the study participants were unable to read and write. Almost half 306(49.8%) of the WDAs were farmers. The majority of 573 (93.2%) of the husbands were farmers. About 313(50.9%) of the study participants have 5–7 families per household (Table 1).

**Table 1:** Socio-demographic characteristics of health development army in central Gondar zone, Northwest, Ethiopia, 2021

Variables	Frequency	Percentage (%)
Age of the HDAs		
18–30	151	24.6
31–45	418	68.0
>=46	46	7.5
**Marital Status**		
Single	31	5.0
Married	531	86.3
Divorced	29	4.7
Windowed	24	3.9
**Educational status of HDAs**		
Unable to read and write	391	63.6
Read and write	148	24.1
Primary education	58	9.4
Secondary and above	18	2.9
**Educational status of Husband**		
Unable to read and write	342	58.6
Read and write	158	27.1
Primary education	51	8.7
Secondary and above	33	5.6
**Occupation of HDAs**		
Housewife	288	46.8
Farmer	306	49.8
Private employee	21	3.4
**Occupation of Husband**		
Farmer	573	93.2
employee	18	2.9
Daily laborer	24	3.9
**Family Size**		
2–4	202	32.8
5–7	313	50.9
>=8	100	16.3

### Level of knowledge of health development army on GMP

Overall, More than two-thirds 440(71.5%) of the study participants had sufficient information about GMP service in the local area, while less than half of the women in the development army who participated in this study knew the appropriate age for the children to start GMP. Four hundred sixteen (67.6%) participants of this study correctly responded to the time interval of GMP; however, less than half (40%) of them knew the age group of children who were eligible for GMP service **(**Table 2**).**

**Table 2:** Level of Knowledge of Health development army on GMP at Dembya and Gondar Zuria districts, Central Gondar Zone, North West Ethiopia

Variables	Frequency	Percentage
Knowing about GMP service in the woreda		
Yes	440	71.5
No	175	28.5
**Appropriate age of GMP started**		
At birth	291	47.3
At first month of the child birth	140	22.8
At 45days of the child birth	162	26.3
Others	22	3.6
**Time interval of each GMP**		
Every week	20	3.3
Every two week	3	0.5
Every month	416	67.6
Every three month	128	20.8
Every six month	13	2.1
I don’t know	35	5.7
**Age group of children is eligible for GMP**		
Children under 2 years	318	51.7
Children under 5 years	246	40.0
Sick children at any age	6	1.0
I don’t know	36	6.9
Others	9	1.5

### Proportion of practical contribution of women development army on growth monitoring and promotion

**Fig. 1:**
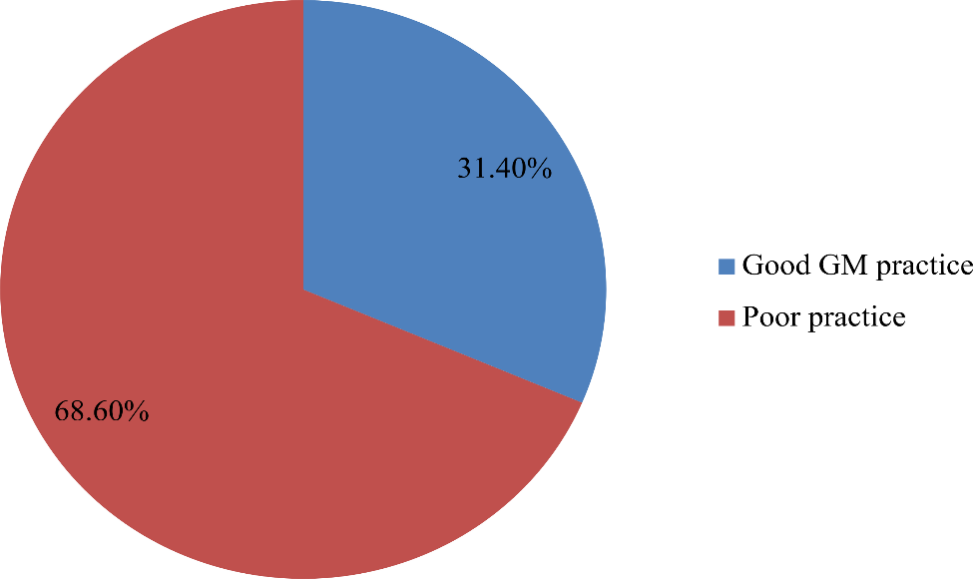
; Proportion of Growth monitoring practice among women development army at Dembya and Gondar Zuria districts, Central Gondar Zone, North West Ethiopia

The overall proportion of growth monitoring practice among Women Development Army in this study was 31.4% (95% CI: 28.0-35.3%) (Fig. 1).

### Factors associated with growth monitoring practice

The result of the multivariate analysis revealed that growth monitoring training, regular community conversation, knowledge of GMP, and a regular schedule of GMP service were found to be statistically and independently associated with the practical contribution of WDA to growth monitoring service in the study area.

Women in the development army who have been trained on growth monitoring were 4 times [AOR = 4.2,95%CI:1.63, 10.58] more likely to contribute to the implementation of growth monitoring compared to those who were not trained, and WDA WDA who had regular community conversations about growth monitoring and community-based nutrition were 6 times [AOR = 3.1,95%CI:3.12, 11.54] more likely to practically contribute to the growth monitoring service in the local area. Similarly, knowledge of growth monitoring was also significantly associated with the practical contribution of WDA to GMP service, that is, women who had good knowledge of GM were two times [AOR = 2.1, 95% CI: 1.17, 3.83] more likely to practise than their counterparts. On the other hand, WDA who worked voluntary in the area where there wasn’t a regular schedule for GMP service were 96% [AOR = 0.04, 95% CI: 0.02, 0.09] less likely to cooperate on GMP services than Kebeles who had a regular schedule for GMP service (Table 3).

**Table 3:** Bivariate and multivariate analysis of factors for practical contribution of Women development Army on growth monitoring and promotion at Dembya and Gondar and Gondar Zuria districts, Central Gondar Zone, North West Ethiopia 2022(n = 615)

Variables	Practical contribution of WDA on GMP		COR (95% CI)	AOR (95% CI)
	Good	Poor		
Age (Year)				
18–30	45	106	0.4(0.18–0.70)	1.2(0.37–3.96)
31–45	123	295	0.4(0.19–0.65)	0.8(0.26–2.25)
>=46	25	21	1	1
Educational status				
Able to read and write and less	157	382	0.5(0.28–0.74)	0.3(1.15–0.81)
Primary education and above	36	40	1	1
Occupational status				
House wife	146	160	5.5(1.58–18.97)	3.9(0.83–8.83)
Farmer	44	244	1.1(0.31–3.83)	0.5(0.11–2.81)
Private employee	3	18	1	1
Regular community conversation				
Yes	118	31	19.8(12.45–31.64)	**6.0(3.12–11.54)**
No	75	391	1	1
GMP training				
No	106	407	1	1
Yes	87	15	22(12.37–40.10)	**4.2(1.63–10.58)**
Knowledge about GMP and CNB				
poor	138	110	1	1
Good	55	312	7.1(4.86–10.41)	**2.1(1.17–3.83)**
Type of position				
1 to 30 leader	105	241	0.9(0.64–1.26)	0.9(0.51–1.61)
1 to 5 leader	88	81	1	
Regular schedule of GMP				
No	68	413		**0.04(0.02–0.09)**
Yes	125	9		1

### Qualitative result

Seven people participated in the in-depth interviews that were held in the local Amharic language. Each interview lasted between forty and sixty minutes. The principal investigator performed the in-depth interview. For a thorough interview, semi-structured, open-ended interviewing guidelines were used.

All of them had at least two years of work experience. Of the seven participants, three were women from the Women Development Army, two were health extension workers, and two were health extension supervisors. Regarding their educational status, two of them were able to read and write, one had primary education, and two of the supervisors were health officers in their profession.

### Barriers of practical cooperation of WDA for growth monitoring service

Most of the study participants reported that the main barrier to cooperation is a lack of awareness about the responsibility of the Women’s Development Army in the area and the benefits of growth monitoring for their children. The programme has received less attention from the health workers and administrative officers.

*A 36years old Women Development Army, ………. “Most of the society thinks that, we are paid for what we do, and when we informed them the schedule, they ignored it. They say that you are working to make money for yourself. Besides, they argued that the child was healthy what was the purpose for measuring his weight at this moment”.*

*A 42 years old Women Development Army, ………“Growth monitoring and promotion service is often as seen as the main package of health extension workers which means it is done together with other packages for instance with vaccination and after the vaccination is over, most of us also not inform them mothers to come on the outreach site to assess the nutritional status of the child.*

*28 years old Health extension workers,……… “Women development army are getting bored because, Even though their work is a volunteer, they are not well supported by training to capacitate them and improve the program*.

*28 years old Health extension supervisor………. “Even though the Women Development Army who are currently working are interested to assist the health extension program. The community conversation which has been held at kebeles level is not consistent and the influential people at the kebeles do not give much help for the women development army.”*

## Discussion

These studies examined that the practical contribution of Women development Army on growth monitoring and promotion service and associated factors at Gondar Zuria and East Dembya districts, northwest Ethiopia. The finding of this study showed that practical contribution of Women Development Army on growth monitoring and promotions service was 31.4% (95% CI: 28.0-35.3%) and training, regular community conversation, knowledge of GMP and regular schedule of GMP service, were independently associated with the outcome variable.

The success of this study was that it was the first that addressed the contribution of WDA to GMP service and applied a mixed study design to create a more context-specific instrument to examine the participants’ lived experience of their contribution to GMP service. Although we attempted to reduce recall bias by interrogating the women, this study has some limitations, like the fact that it excluded the urban population, which may limit the generalizability of its findings.

The findings of this study showed that the practical contribution of the Women Development Army on growth monitoring and promotion was 31.4% (95% CI: 28.0–35.3%), which was in line with their contribution on institutional delivery (28%) [31, 32]. However, it was lower than their involvement in antenatal care visits (51%), [33]. The disparity might be due to the fact that the frequency of growth monitoring and promotion has been every month, but ANC visits has every 2-3 months that might not made challenging on the contribution of woman development army furthermore, growth monitoring has been implemented on the healthy children which might have less attention by the mothers or care givers compared with ANC service.

Training in growth monitoring and community-based nutrition was an important positive predictor of good practise and the contribution of women development army compared to those who were not trained. The possible explanation might be that training about basic information on growth monitoring and promotion would improve the knowledge and practise of women development army. In addition, refreshment training would motivate them to contribute to the implementation of GMP in their local area [34].

WDA who had regular community conversations about growth monitoring and community-based nutrition were more likely to implement growth monitoring in the local area. The probable explanation might be that regular meetings with the community are a good opportunity to discuss with them how to solve the problem in time and explain the benefits of growth monitoring for the child to improve their health and nutritional status. This finding was supported by the qualitative report. A 34 years women development army *……… “If there is a need to improve growth monitoring program, it is necessary to strengthen the community conversation because it is a platform where to correct the gaps and share our experience to other women development member….”.*

Knowledge of women development army on growth monitoring and community based nutrition was also significantly associated with GMP practical contribution. WDA who had good knowledge of GM were three times more likely to practise than their counterparts. The reason might be the fact that knowledge is the foundation of all actions. For the most part, knowledge and related skills were necessary to practise and assist health extension workers in various forms of healthcare activities in the local area. Furthermore, it may have a positive effect to acquire procedural skills to perform and assist the health extension workers in the implementation of growth monitoring and promotion in the local area.

Similarly, a regular schedule of growth monitoring and community based nutrition service in the local area was significantly associated with good practise in growth monitoring service in the area. That is, WDA who worked voluntary in the area where there wasn’t a regular schedule for GMP service were 96% less likely to cooperate on GMP services than Kebeles who had a regular schedule of GMP service. The probable reason might be the fact that the women who have worked in the area were not employed for the service but provided voluntary service for the programme and had their own usual activities; therefore, if the schedule is not reliable, they will be discouraged and unwilling to cooperate for the growth monitoring service. This finding was supported by the qualitative result. A 41 years old women development army *“…….We always inform the mothers a day early to attend the growth monitoring and promotion program, but after scheduled them to attend timely, the program is cancelled because of the health extension workers are assigned to work on another urgent campaign and this hinders us from doing our job properly*.

## Conclusion

The WDA structure’s practical impact on GMP service in this study was minimal. The practical value of the growth monitoring service was strongly correlated with GMP training, regular community conversation, knowledge, and a regular schedule of GMP service in the community. They did not effectively contribute to GMP service due to a lack of training, low motivation among WDA, and poor communication between WDA and health extension workers. Therefore, providing WDA training and enhancing community discourse at the kebeles level are crucial to achieving the WDA structure on GMP services.

## Data Availability

Full data set and materials pertaining to this study can be obtained from corresponding author on reasonable request.

## Abbreviations

AOR: Adjusted Odd Ratio
CBN: Community Based Nutrition
COR: Crude Odd Ratio
EDHS: Ethiopian Demographic and Health Survey
GMP: Growth Monitoring and promotion
HEP: Health Extension packages
HEW: Health Extension Workers
UNICEF: United Nation International Children’s Fund
WDA: Women Development Army
WDTs: Woman Development Teams
WHO: World Health Organization.

## References

[1] Lee BX (2016). Transforming our world: implementing the 2030 agenda through sustainable development goal indicators. J Public Health Policy.

[2] Organization WH. *UNICEF/WHO/The World Bank Group joint child malnutrition estimates: levels and trends in child malnutrition: key findings of the 2020 edition* 2020.

[3] Organization WH. Global status report on alcohol and health 2018. World Health Organization; 2019.

[4] Organization WH (2014). Children: reducing mortality. Wkly Epidemiol Record = Relevé épidémiologique hebdomadaire.

[5] Brabin BJ, Coulter JB. Nutrition-associated disease, Manson’s tropical diseases. 2009, Elsevier. 537–55.

[6] Lu C, Black MM, Richter LM (2016). Risk of poor development in young children in low-income and middle-income countries: an estimation and analysis at the global, regional, and country level. The Lancet Global Health.

[7] McCoy DC (2017). Correction: early Childhood Developmental Status in Low-and Middle-Income Countries: National, Regional, and global prevalence estimates using predictive modelling. PLoS Med.

[8] Organization WH. *Levels and trends in child malnutrition: UNICEF* 2021.

[9] Soliman A et al. *Early and long-term consequences of nutritional stunting: from childhood to adulthood*. Acta Bio Medica: Atenei Parmensis, 2021. 92(1). 10.23750/abm.v92i1.11346 PMC7975963 33682846

[10] Dewey KG, Begum K (2011). Long-term consequences of stunting in early life. Matern Child Nutr.

[11] Black RE (2008). Maternal and child undernutrition: global and regional exposures and health consequences. The lancet.

[12] Wang H (2016). Global, regional, national, and selected subnational levels of stillbirths, neonatal, infant, and under-5 mortality, 1980–2015: a systematic analysis for the global burden of Disease Study 2015. The Lancet.

[13] Victora CG (2008). Maternal and child undernutrition: consequences for adult health and human capital. The lancet.

[14] Food. In: Nations U, editor. The State of Food and Agriculture 2013: Food Systems for Better Nutrition. Food and Agriculture Organization of the United Nations; 2013. and A.O.o.t.

[15] Baye K, Laillou A, Chitweke S (2020). Socio-economic inequalities in child stunting reduction in sub-saharan Africa. Nutrients.

[16] da Silva ICM (2018). Socioeconomic inequalities persist despite declining stunting prevalence in low-and middle-income countries. J Nutr.

[17] Ethiopia. FDRo. Mini demographic and Health Survey 2019 Key indicators. Federal Ministry of Health and Ethiopian Public Health Institute and Addis Ababa; 2019.

[18] Ethiopia FDRo. *The Social and Economic Impact of Child Undernutrition in Ethiopia Summary Report*. 2009.

[19] Black RE (2013). Maternal and child undernutrition and overweight in low-income and middle-income countries. The lancet.

[20] Dukhi N. *Global Prevalence of Malnutrition: Evidence from Literature*, in *Malnutrition*. 2020, IntechOpen.

[21] Griffiths M, Rosso JD (2007). Growth monitoring and the promotion of healthy young child growth: evidence of effectiveness and potential to prevent malnutrition.

[22] Tremlett G, Lovel H, Morley D. Guidelines for the design of national weight-for-age growth charts. UNICEF; 1983.

[23] Bhutta ZA (2010). Global experience of community health workers for delivery of health related millennium development goals: a systematic review, country case studies, and recommendations for integration into national health systems. Global health workforce Alliance.

[24] Damtew ZA (2018). Correlates of the women’s Development Army strategy implementation strength with household reproductive, maternal, newborn and child healthcare practices: a cross-sectional study in four regions of Ethiopia. BMC Pregnancy Childbirth.

[25] Sudhakar M et al. *Primary care systems profiles and performance (PRIMASYS): ethiopian case study*. Alliance Heal Policy Syst Res, 2017: p. 18–25.

[26] Mangasaryan N, Arabi M, Schultink W (2011). Revisiting the concept of growth monitoring and its possible role in community-based nutrition programs. FoodNutr Bull.

[27] Kebede GG, Dawed YA, Seid KA (2022). Child growth monitoring and promotion practice and associated factors among health care workers at public health facilities in south Wollo Zone, Northeast Ethiopia: a facility-based cross-sectional study. BMC Nutr.

[28] Ashworth A, Shrimpton R, Jamil K (2008). Growth monitoring and promotion: review of evidence of impact. Matern Child Nutr.

[29] Agency CS. Ethiopian demographic and Health Survey report. Addis Ababa,Ethiopia; 2011.

[30] Agency CS. Ethiopian demographic and Health Survey report. Addis ababa,Ethiopia; 2016.

[31] Yitbarek K, Abraham G, Morankar S (2019). Contribution of women’s development army to maternal and child health in Ethiopia: a systematic review of evidence. BMJ open.

[32] Aregawi HG (2017). Determinants of defaulting from completion of child immunization in Laelay Adiabo District, Tigray Region, Northern Ethiopia: a case-control study. PLoS ONE.

[33] Girmaye M, Berhan Y (2016). Skilled antenatal care service utilization and its association with the characteristics of women’s health development team in Yeky District, south-west ethiopia: a multilevel analysis. Ethiop J Health Sci.

[34] Hatlevik IKR (2012). The theory-practice relationship: reflective skills and theoretical knowledge as key factors in bridging the gap between theory and practice in initial nursing education. J Adv Nurs.

